# Opportunities and challenges for modelling epidemiological and evolutionary dynamics in a multihost, multiparasite system: Zoonotic hybrid schistosomiasis in West Africa

**DOI:** 10.1111/eva.12529

**Published:** 2017-09-09

**Authors:** Anna Borlase, Joanne P. Webster, James W. Rudge

**Affiliations:** ^1^ Department of Pathobiology and Population Sciences Centre for Emerging, Endemic and Exotic Diseases Royal Veterinary College University of London London UK; ^2^ Department of Infectious Disease Epidemiology London Centre for Neglected Tropical Disease Research School of Public Health Imperial College London London UK; ^3^ Communicable Diseases Policy Research Group London School of Hygiene and Tropical Medicine London UK; ^4^ Faculty of Public Health Mahidol University Bangkok Thailand

**Keywords:** evolution, hybridization, mathematical modelling, multihost, multiparasite, *R*_0_, reservoir, *Schistosoma* spp., spillover, zoonoses

## Abstract

Multihost multiparasite systems are evolutionarily and ecologically dynamic, which presents substantial trans‐disciplinary challenges for elucidating their epidemiology and designing appropriate control. Evidence for hybridizations and introgressions between parasite species is gathering, in part in line with improvements in molecular diagnostics and genome sequencing. One major system where this is becoming apparent is within the Genus *Schistosoma*, where schistosomiasis represents a disease of considerable medical and veterinary importance, the greatest burden of which occurs in sub‐Saharan Africa. Interspecific hybridizations and introgressions bring an increased level of complexity over and above that already inherent within multihost, multiparasite systems, also representing an additional source of genetic variation that can drive evolution. This has the potential for profound implications for the control of parasitic diseases, including, but not exclusive to, widening host range, increased transmission potential and altered responses to drug therapy. Here, we present the challenging case example of haematobium group *Schistosoma* spp. hybrids in West Africa, a system involving multiple interacting parasites and multiple definitive hosts, in a region where zoonotic reservoirs of schistosomiasis were not previously considered to be of importance. We consider how existing mathematical model frameworks for schistosome transmission could be expanded and adapted to zoonotic hybrid systems, exploring how such model frameworks can utilize molecular and epidemiological data, as well as the complexities and challenges this presents. We also highlight the opportunities and value such mathematical models could bring to this and a range of similar multihost, multi and cross‐hybridizing parasites systems in our changing world.

## 1 INTRODUCTION

Hybridization of parasites represents an emerging public health concern with the potential for significant impact on parasite evolution and epidemiology worldwide and posing diverse challenges for the control of parasitic diseases (King, Stelkens, Webster, Smith, & Brockhurst, 2015). Human migration, changing agricultural practices and climate change all contribute to an increased potential for human and animal populations to encounter new infectious agents and for the increased incidence of co‐infection by multiple parasite species and strains within the same host (Astrom et al., 2012; Bett et al., 2017; Cable et al., 2017; Castelli & Sulis, 2017; Jones, Brophy, Mitchell, & Williams, 2017; Shragai, Tesla, Murdock, & Harrington, 2017). Such multiparasite systems are of particular evolutionary and epidemiological interest due to the potential for complex interactions, including competition and/or synergies between parasites within the same host (Nowak & May, 1994; Pedersen & Fenton, 2007; Petney & Andrews, 1998). In certain circumstances, such mixed species co‐infections can allow for heterospecific (between‐species or between‐lineage) mate pairings (Detwiler & Criscione, 2010; King et al., 2015).

Recent developments in molecular tools have revealed many examples of cross‐species pairings leading to viable hybrids (hybrids defined as offspring resulting from the interbreeding between two species) and introgressions (the introduction of single genes or chromosomal regions from one species into another through repeated backcrossing) in parasites of both animals and human populations (Criscione et al., 2007; Grigg, Bonnefoy, Hehl, Suzuki, & Boothroyd, 2001; Le et al., 2008; Ravel et al., 2006; Webster, Diaw, Seye, Webster, & Rollinson, 2013). Hybridizations and introgressions represent an additional source of genetic variation that may drive parasite evolution, with the potential for, amongst others, increased host range, altered pathology and resistance to drug therapy (King et al., 2015). Important examples include the naturally occurring hybridizations between *Leishmania infantum* and *Leishmania major* (Ravel et al., 2006), which have been observed to exhibit enhanced transmission potential and host range (Volf et al., 2007), and within *Trypanosoma brucei* subspecies, where introgressions have been shown to be associated with increased virulence and host range (Echodu et al., 2015; Goodhead et al., 2013).

There is a growing array of examples of naturally occurring hybridizations between *Schistosoma* species across sub‐Saharan Africa (reviewed in Léger & Webster, 2017). Of particular interest in terms of parasite evolution, epidemiology and control is the confirmation of widespread viable hybridizations between the human schistosome species *Schistosoma haematobium* and the livestock schistosome species *S. bovis* and/or *S. curassoni* in West Africa (Huyse et al., 2009; Webster et al., 2013), and between *S. haematobium* and the livestock schistosome species *S. mattheei* in Southern Africa (De Bont, Vercruysse, Southgate, Rollinson, & Kaukas, 1994; Vercruysse et al., 1994). These examples are of profound significance as they point towards the zoonotic potential of this group of parasites within Africa, indicating that, at least in some settings in Africa, schistosomiasis transmission may represent a complex multihost as well as multiparasite system.

Mathematical models have been used for many years to gain insights into the complex processes underlying the transmission dynamics of infectious diseases (Grassly & Fraser, 2008; Heesterbeek & Roberts, 1995), including schistosomiasis (Anderson & May, 1991; Anderson, Turner, Farrell, Yang, & Truscott, 2015; Gurarie, King, Yoon, & Li, 2016). There are many examples where mathematical models have played a crucial role in informing policy, optimizing control methods and driving research questions (Luz, Vanni, Medlock, Paltiel, & Galvani, 2011; Rivers, Lofgren, Marathe, Eubank, & Lewis, 2014; Turner, Walker, Churcher, & Basáñez, 2014), and the development and improved application of mathematical models has been identified as a priority for the facilitation of progress towards the control and elimination of human helminthic diseases (Basáñez et al., 2012; Boatin et al., 2012; Utzinger, 2012). Furthermore, modelling frameworks have been instrumental in determining the role of animal reservoirs in multihost systems (Fenton, Streicker, Petchey, & Pedersen, 2015; Funk, Nishiura, Heesterbeek, Edmunds, & Checchi, 2013; Rudge et al., 2013).

There is a growing recognition of the need for mathematical modelling approaches to consider the impact of evolutionary processes on epidemiological dynamics, and synthesize data from across disciplines and scales, from the molecular to the population level (Gandon, Day, Metcalf, & Grenfell, 2016; Gog et al., 2015; Metcalf et al., 2015). Examples of the application of such approaches include the prediction of *Mycobacterium tuberculosis* drug resistance evolution under different treatment strategies (Luciani, Sisson, Jiang, Francis, & Tanaka, 2009) and the optimization of vaccine design using predictions of seasonal influenza antigenic evolution (Neher, Bedford, Daniels, Russell, & Shraiman, 2016).

The identification of hybridized *Schistosoma* systems in West Africa raises many important research questions. These include the need to elucidate the relative importance of the different potential host species and the interactions between the *Schistosoma* species, and to investigate the potential for anthelmintic resistance to develop and establish, thereby identifying if and how current control strategies may need to be modified in hybrid zones. Mathematical model frameworks representing this complex multiparasite, multihost system, parameterized and fitted to molecularly characterized field data, could prove crucial to answering many of these questions as well as potentially being applicable to other hybrid systems. However, to the authors’ knowledge, there are no current mathematical models relating directly to mixed species hybridizations and introgressions within any multihost system.

**Figure 1:**
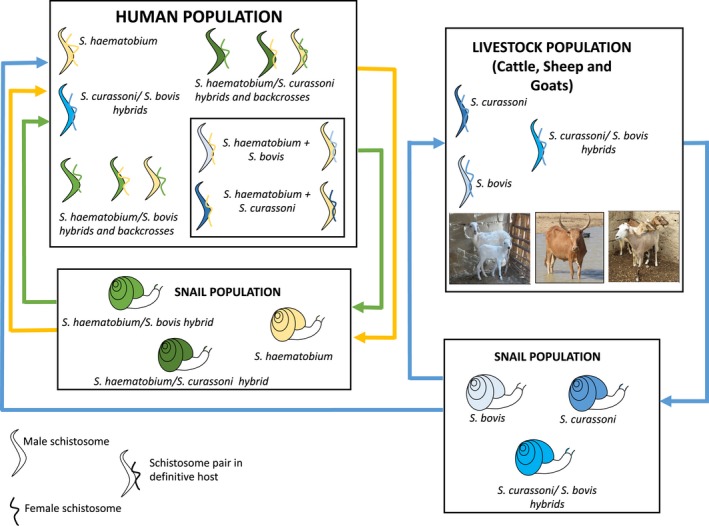
The zoonotic hybrid schistosome system in West Africa, haematobium group. Summarized knowledge of schistosome species and hybrids within the haematobium group in the human and livestock population of West Africa is depicted in this schematic. Human schistosome species are shown as yellow, animal schistosome species are shown as blue, and hybrids between animal and human schistosome species are shown as green, with infected snails also represented with corresponding colours

Using the case example of zoonotic hybrid schistosomes of the haematobium group in West Africa (outlined in Figure 1), we explore here the challenges of modelling an evolving multihost multiparasite system. We identify data gaps and consider the questions raised and ways in which they may be addressed, as well as the potential opportunities and value mathematical models may bring for gaining insights relevant to control of schistosomiasis in a rapidly changing world.

## 2 EVIDENCE OF ZOONOTIC HYBRID SCHISTOSOMES IN WEST AFRICA AND POTENTIAL IMPLICATIONS FOR TRANSMISSION AND CONTROL

**Figure 2:**
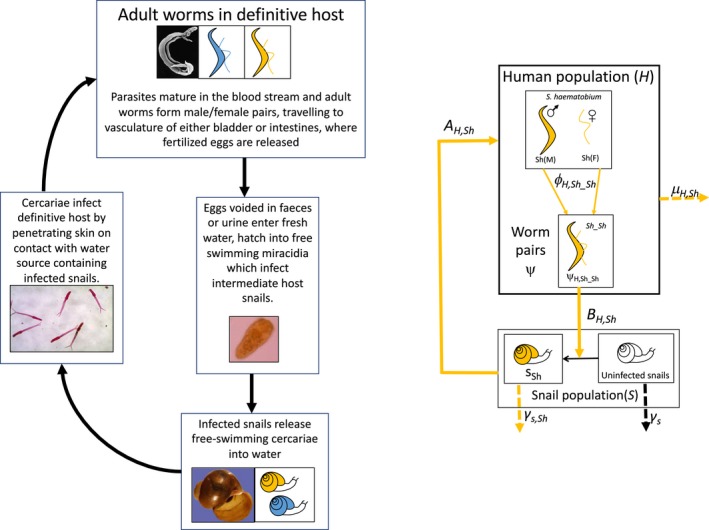
The life cycle of the schistosome parasite and a generalized framework for a *Schistosoma haematobium* transmission model. Definitions of example parameters and factors influencing parameters given in Table 1

Schistosomiasis, caused by parasites within the dioecious *Schistosoma* genus of helminths, represents a significant disease burden to both humans and animals in sub‐Saharan Africa (Colley, Bustinduy, Secor, & King, 2014; De Bont & Vercruysse, 1997; Vercruysse, Southgate, & Rollinson, 1985; Gower, Vince, & Webster, 2017). Schistosomes have a complex life cycle involving a definitive mammalian host, with transmission mediated by a freshwater snail intermediate host (outlined in Figure 2). *Schistosoma haematobium*, which causes urogenital schistosomiasis in humans, is endemic in many countries across sub‐Saharan Africa, including Senegal and Niger within West Africa (Knowles et al., 2015). Infection typically causes haematuria and pain on urination, and has many serious potential long‐term consequences, including fibrosis of the urinary tract, renal dysfunction and an increased risk of bladder cancer (Colley et al., 2014). The closely phylogenetically related livestock schistosome species *S. bovis* and *S. curassoni,* both within the larger haematobium group, are also present in West Africa. These are intestinal schistosomes which can infect cattle, sheep and goats, potentially causing enteritis, anaemia, emaciation and even death in these species (De Bont & Vercruysse, 1997; Vercruysse et al., 1985).

The molecular studies of schistosomes obtained from human definitive hosts and molluscan (*Bulinus truncatus* and *B. globosus*) intermediate hosts in Senegal and Niger (Huyse et al., 2009; Webster et al., 2013) which confirmed hybridization between animal and human schistosomes within the haematobium group, specifically identified hybrids of *S. haematobium* with *S. bovis*, and/or with *S. curassoni,* and complementary laboratory passage of parasites demonstrated that these hybrids were viable and fertile (Webster et al., 2013). Importantly, multilocus techniques identified putative “first‐generation‐type” or F1‐type hybrids amongst the schistosome eggs excreted by humans, indicative of initial cross‐species pairings between “pure” *S. haematobium* worms and zoonotically acquired livestock schistosome species. In addition, molecular data indicated that hybridization was bidirectional (where males and females of each species were able to pair and produce viable offspring within the human host) and identified the presence of introgressed hybrids (due to backcrosses between hybrids and parent species). Furthermore, studies in Niger have found that hybrids of *S. bovis* with *S. curassoni*, previously identified in the livestock population in several countries in West Africa (Rollinson, Southgate, Vercruysse, & Moore, 1990; Webster et al., 2013), can cause patent infections in humans (Léger et al., 2016), providing additional evidence for the potential importance of livestock as a source of infection to humans.

All of these findings are of particular significance as livestock schistosome reservoirs had not traditionally been considered of importance within Africa in general, and *S. haematobium* in particular was thought to be maintained solely within the human population. This is in contrast to the major schistosome species in Asia (*S. japonicum* and *S. mekongi*), which are known zoonoses, with natural infections found across multiple species of mammalian hosts. Accordingly, animal *Schistosoma* spp. in Africa were considered of little or no public health significance, and there have been relatively very few studies in livestock or wildlife. Indeed, to the authors’ knowledge, there are no publicly available recent surveillance data on schistosomiasis in the livestock of West Africa (Gower, Vince et al., 2017). To date, no hybrids between *S. haematobium* with *S. bovis* or *S. curassoni* have been identified in any animal populations. However, previous studies have been cross‐sectional and focused on abattoir carcasses, with no live animal sampling, nor any urinary/urogenital tract sampling (where such hybrids may be predicted to have a predilection (Cunin, Tchuem Tchuente, Poste, Djibrilla, & Martin, 2003; Koukounari et al., 2010)). (Ongoing work in West Africa is, however, seeking to determine whether human–animal schistosome hybrids are present in the livestock population, and crucially, whether the initial cross‐species pairings may be occurring in the livestock species. (http://www.gla.ac.uk/researchinstitutes/bahcm/zels-as/about_zels/)).

There are many possible implications for schistosome hybridization of relevance for transmission and control. In addition to the potential for a widened definitive host range, there is also evidence that hybrid schistosomes may have an increased range of intermediate snail hosts relative to their pure “parent” single species (Huyse et al., 2009; Morgan et al., 2003; Southgate, van Wijk, & Wright, 1976; Wright & Ross, 1980), which may also enable a wider geographical range for hybrid schistosome infections. A recent outbreak of urogenital schistosomiasis in Corsica (in which humans were found to be infected with *S. haematobium* and *S. haematobium* with *S. bovis* hybrids) has demonstrated the potential for migration to introduce hybrid schistosomes into new areas where suitable snail intermediate hosts are found (Boissier et al., 2016). Of additional concern is the potential for hybrids to have traits associated with increased “fitness” (heterosis or hybrid vigour). This has been observed in experimental studies of schistosome hybrids within laboratory rodents, including those of *S. haematobium* with *S. guineensis,* and *S. haematobium* with *S. mattheei* (Taylor, 1970; Webster & Southgate, 2003), where hybrids displayed properties associated with increased fitness at multiple stages of the life cycle, including increased adult worm fecundity and increased cercarial shedding rates in snails (Taylor, 1970; Webster & Southgate, 2003).

Preventative chemotherapy of school‐aged children represents the mainstay of current antischistosomiasis control programmes in sub‐Saharan Africa, with praziquantel presently the only available drug (Webster, Molyneux, Hotez, & Fenwick, 2014). Resistance to praziquantel has not yet appeared to be a significant problem in the human population (Fenwick, 2015), but there is concern that hybridization could potentiate evolution of drug resistance, with known examples in parasite and rodent species of drug‐resistance genes being introgressed into new populations through hybridization (Chaudhry et al., 2015; Song et al., 2011). Conversely however, hybridization and a corresponding widening host range may lead to *refugia* for drug‐susceptible genotypes and thus potentially help maintain drug susceptibility (King et al., 2015; Webster, Gower, Knowles, Molyneux, & Fenton, 2016; Gower, Vince et al., 2017).

Mathematical modelling provides a crucial tool for exploring and understanding the potential implications of hybridization for the transmission and control of schistosomiasis. In the following sections, we present an overview of schistosomiasis transmission models to date and identify key challenges and opportunities for extending such frameworks to consider multiple host species and parasite hybridizations.

## 3 MATHEMATICAL MODELS OF SCHISTOSOME TRANSMISSION DYNAMICS

Mathematical models of parasite transmission generally aim to describe and understand the dynamics of parasite populations with respect to time, incorporating those aspects of the parasite life cycle relevant to the specific research question and, for macroparasites, often encompassing both prevalence and infection intensity in definitive hosts (Basáñez et al., 2012). Key aspects of the *Schistosoma* spp. life cycle are summarized in Figure 2, alongside a schematic representing a generic model of *S. haematobium* transmission, acknowledging that not all aspects or stages of the transmission cycle may necessarily need to be incorporated explicitly into a model of schistosomiasis transmission. Example parameters and some of the factors influencing them are identified in Table 1. Density‐dependent processes, both positive (for example, the increased probability of worms mating as the number of worms increases within a host) and negative (for example, reduced fecundity of worm pairs as the number of worms in a host increases), play a key role in the *Schistosoma* spp. system, with models taking differing approaches to incorporating these effects.

**Table 1:** Parameters and variables for hybrid schistosome system outlined in Figures 2 and 3 and key factors that may influence them

Parameter/Variable	Description	Notes/Factors influencing
*H*	Human population	Birth rate, death rate, immigration, migration
*C*	Cattle population	Birth rate, death rate, immigration, migration
*G*	Goat population	Birth rate, death rate, immigration, migration
*O*	Sheep population	Birth rate, death rate, immigration, migration
*S*	Snail population	Snail habitat, natural lifespan of snails, interventions targeting snail population
*m*_*i,j*_	Mean number of worms in definitive host *i*, of schistosome species *j*	Definitive host *i* can be: *H *= human, *C *= cattle, *G *= goats, *O = *sheep. Schistosome species *j* can be: *Sh* = *S. haematobium Sb* = *S. bovis* *Hyb* = Hybrid of *S. haematobium* and *S. bovis. m*_*i,j*_ and κ_*i,j*_ determined by number and dispersion of worms in the definitive host populations
κ_*i,j*_	Clumping parameter for distribution of schistosome species *j* in host species *i*
*q*_*i,j*_	Proportion of females of schistosome species *j* in host *i*	
ϕ_*i,j_k*_	Probability that a female of species *j* will form a pair with male of species *k* in host species *i*	Function of *m* and κ and *q*
ψ_*i,j_k*_	Total number of couples of pairing *j_k* in host species *i*	
*s*_*j*_	Number of snails shedding cercariae of species *j*	
*B*_*i,j*_	Transmission rate of schistosome species *j* from definitive host species *i* to snails	Determined by worm pair fecundity, contact rate of definitive host with water, miracidia viability and probability of miracidia penetrating snail
*A*_*i,j*_	Transmission rate of schistosome species *j* from snails to definitive host species *i*	Determined by contact rate of definitive host with snail habitats, cercariae‐shedding rate, probability of cercariae penetration
*μ*_*i,j*_	Death rate of mature schistosomes of species *j* in host species *i*	Natural lifespan of schistosomes, death rate due to interventions such as praziquantel treatment: efficacy and frequency of drug treatment
*γ*_*s*_ *(γ*_*s,j*_ *)*	Death rate of snails (death rate of snails infected with schistosome species *j*)	Seasonality, habitat, (infection status)

The first schistosome transmission model described by Macdonald (1965) represented a deterministic framework tracking total number of adult female parasites in the entire population of definitive hosts and the number of infected snails. In this framework, the intermediate snail host alone represented the density‐dependent stage; that is, it is assumed that snails can only get infected once and produce cercariae at constant rate, meaning that at higher worm population sizes the contribution of individual worms to transmission is less. This is a common simplifying assumption in schistosome models, although it is now known that snails can have mixed infections, including evidence of single snails shedding both hybrid and pure species (Davies, Fairbrother, & Webster, 2002; Léger, E. & Webster, J.P. unpublished).

May (1977) developed the worm burden model further to consider sexual reproduction within the schistosome life cycle, incorporating the aggregation of adult worms across the definitive host population, and their probability of mating. A negative binomial function was applied to represent the distribution of worms using two parameters: mean number of worms per host, *m*, and a “clumping” parameter, κ, with a smaller κ representing increasing overdispersion and an increasing κ moving towards a Poisson distribution with worms randomly distributed amongst the host population. This framework has been further adapted to consider different assumptions regarding mating behaviour and sex ratios (May & Woolhouse, 1993), density‐dependent decrease in worm fecundity (Medley & Anderson, 1985) and various heterogeneities, such as age structure, in the definitive host population (Anderson et al., 2015; Chan et al., 1995; Liang, Spear, Seto, Hubbard, & Qiu, 2005).

A key concept in infectious disease epidemiology is the basic reproduction number, *R*_0_, which gives a threshold for a parasite to spread within population (*R*_0_ > 1), and mathematical transmission models can provide a tool for describing and estimating *R*_0_. In worm burden models, *R*_0_ can be defined as the average number of mated female schistosomes produced during the lifetime of a single mated female schistosome, in the absence of density‐dependent constraints on parasite establishment, survival or reproduction (Anderson & May, 1991). Barbour (1996) argued that such frameworks underestimate *R*_0_ due to insufficient consideration of density dependence at the definitive host stage, and described an alternative, prevalence‐type model based on the Ross malaria model (Ross, 1905). Here, both intermediate and definitive hosts are classified only as infected or uninfected. In such prevalence‐based frameworks, more similar to other transmissible infections, *R*_0_ is interpreted as the average number of infected individual definitive hosts resulting from a single infected definitive host in an otherwise uninfected population.

An advantage of the Barbour‐type model is that it can be fitted to prevalence data, which can be directly measured in a population, unlike schistosome worm burden which is usually estimated based on an assumed relationship between measured egg outputs and intensity of infection in the definitive host. Barbour also argued that modelling the acquisition of “infected status” in one instantaneous “clump” can give a better fit to the observation of increasing prevalence with age, compared with the gradual accumulation of worms over time through a “trickle” of infections that is typically assumed in a worm burden model. However, as prevalence frameworks do not track the number or distribution of worms amongst the host population, they cannot easily incorporate the impact of these dynamic variables on important factors such as schistosome mating probabilities, definitive host recovery rates and susceptibility to re‐infection.

Individual or agent‐based models have also been described for schistosomiasis (Hu, Gong, & Xu, 2010; Mitchell, Mutapi, Savill, & Woolhouse, 2012; Wang & Spear, 2015), tracking individuals in the population of interest which can enable better representation of heterogeneities in factors such as sex, immunity, spatial factors and host interactions with the environment at the individual level. However, such models can be very computationally intensive, especially for large populations. Moreover, the increase in realism that can (in principle) be gained through more complex models comes at a cost of reduced analytical tractability and mechanistic understanding of how specific processes may govern transmission dynamics.

## 4 CONSIDERATIONS FOR DEVELOPING AND PARAMETERIZING A TRANSMISSION MODEL OF THE INTERSPECIFIC SCHISTOSOME HYBRID SYSTEM

**Figure 3:**
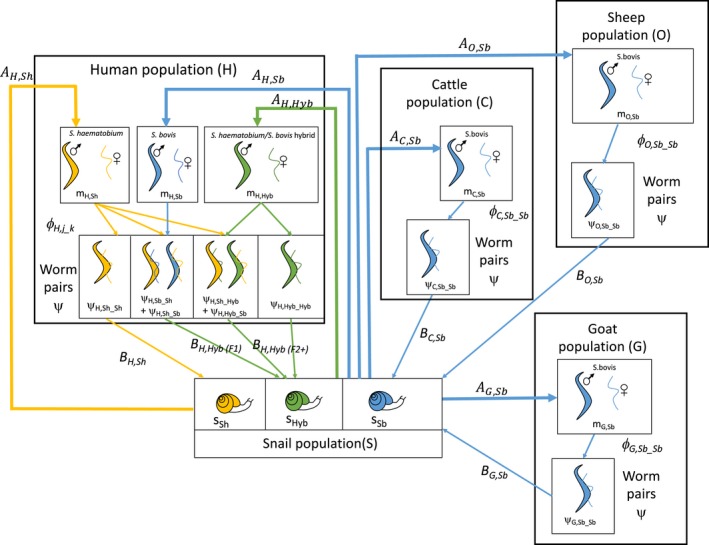
A preliminary framework for a multihost, multiparasite transmission model of the zoonotic hybrid *S. haematobium/S. bovis* system, involving cattle, sheep, goats and humans definitive hosts. Definitions of example parameters and factors influencing these parameters are given in Table 1. For clarity, the “worm death rate” parameter *μ*_*i,j*_ and the “snail death rate” parameters *γ*_*s,j*_ are not shown but would be included, and likewise all the possible mating probabilities for female worms in the human host population are not shown (*ϕ*_*i,j_j*_), but would all need to be included in such a model

Designing and parameterizing a model of the haematobium group hybrid system described in Section 4 poses many challenges, which can be broadly categorized based on their associations with the following: (i) parasite interactions in this multipathogen system; (ii) multiple definitive hosts involved in this system; and (iii) unknown and potentially evolving properties of the parasite population. With reference to Figure 3, which presents a preliminary framework of a hybrid system model involving multiple types of definitive hosts (cattle, sheep, goats and humans) and parasites (*S. bovis*,* S. haematobium*, and their hybrids), these challenges and the possible approaches to tackling them will now be discussed.

### 4.1 Parasite interactions: hybrid schistosomes as a multipathogen system

When designing a model of a multiparasite system, one of the first considerations is how to define the various parasites in order to allow for heterogeneities and interactions between them. In the preliminary framework given in Figure 3, we differentiate between three types of schistosomes: *S. haematobium* (*Sh*), *S. bovis* (*Sb*) and their hybrids (*Hyb*). For simplicity, we are excluding the livestock schistosome *S. curassoni* and its hybrids in the example framework. In this multihost system, one must next consider in which host species each of these parasite types can occur. In the absence of any current evidence for *S. haematobium* or its hybrids in livestock populations, we assume here that livestock are susceptible only to *S. bovis*. It is assumed humans can be infected with *S. haematobium* and its hybrids; however, patent *S. bovis* infections have not been observed in humans in West Africa (although evidence for patent *S. bovis:S. bovis* pairings in human hosts was described in the Corsica outbreak, see Boissier et al., 2016). Given the evidence for F1‐type *S. haematobium* with *S. bovis* hybrids in the human population, it is therefore assumed in this framework that *S. bovis* can infect humans but only cause patent infection when paired with *S. haematobium*. Uncertainties around all these assumptions are discussed later.

The next consideration for model design is how to best incorporate the primary interactions of interest between the different parasites. In a schistosome hybrid system, the initial cross‐species pairings between animal and human schistosomes is of particular significance, and arguably the most complex and challenging aspect of the system to model. As illustrated in Figure 3, these parings require three key conditions to be met: firstly, transmission of one schistosome species (*Sb*, in our example) from its “normal” reservoir or maintenance definitive hosts (livestock) into the “spillover” or nonmaintenance definitive host species (humans), so that both the livestock and human schistosomes are simultaneously present in the same definitive host group. This process relates to the multihost nature of the system, and the modelling considerations associated with this are discussed further in the next section. Secondly, schistosomes of both species must be simultaneously present in an individual host. Finally, parasites of the different species must form male–female pairs, to generate the viable hybrid parasite population, the probability of which will also depend on mating behaviour and sex ratios. As illustrated in Figure 3, there is then the potential for the hybrid offspring of these cross‐species pairings to be propagated in the human host population, with onward transmission and subsequent mating with one of their parental species (introgression) or with other hybrids. Assuming the three distinct parasite types within the human host population defined above, there are six potential mating combinations, or nine permutations if one also differentiates between the sex of each parasite within the pairing (which may be important if there are unequal sex ratios). Our preliminary framework (Figure 3) depicts only four of the potential mating combinations in the human hosts indicated by current molecular evidence from West Africa (*Sh:Sh, Sh:Sb, Sh:Hyb* and *Hyb:Hyb*), but in reality, and taking into account the additional possibility of co‐infection with *S. curassoni*, the potential mating combinations are likely to be even more complex.

Clearly, the need to consider the probabilities of various potential mating combinations is of central importance to designing a model of the zoonotic hybrid schistosome system. As these probabilities will depend on the number and distribution of worms for each parasite species in the host populations, which are not incorporated into prevalence frameworks, one intuitively looks towards worm burden models as a more suitable approach for modelling such a system.

In a mean worm burden model, the force of infection acting on the snail population is dependent on the number of mated pairs (*ψ*), which in turn depends on the number of females and the probability that a female is mated, *ϕ*_._ Adapting these to our preliminary multihost, multischistosome framework (Figure 3 *), ψ*_*i,j_k*_ and *ϕ*_*i,j_k*_ *,* represent the number of worm pairs and mating probability of a female schistosome of species *j* with a male of species *k*, within definitive host group *i*. The mating probability itself is typically modelled as a function of the mean worm burden, *m*, the clumping parameter, κ*,* and the proportion of schistosomes that are female, *q*, although the precise formulation of this function depends on assumptions regarding parasite mating behaviour (e.g., monogamy vs. polygamy) (May & Woolhouse, 1993). In the zoonotic hybrid system, one must also consider potential heterogeneities in each of these factors across parasite (and host) species.

As worm burden cannot be directly measured in human hosts, worm burden per host is usually estimated by model fitting to egg‐count data, with a functional relationship between egg count and worm burden assumed. For schistosomiasis in humans, this assumed relationship has typically been based on a few historical autopsy studies (Cheever, 1968; Cheever, Kamel, Elwi, Mosimann, & Danner, 1977) which has many limitations. However, in hybrid zones where many people are likely to be co‐infected with pure species and hybrids, fitting egg output data to a model incorporating pairings between different schistosome species and estimating the number of worm pairs per hosts will require differentiation between the progeny of co‐existing species. Furthermore, different pairings may result in different fecundity rates. This poses significant challenges, but molecular approaches such as parentage and/or sib‐ship analysis of schistosome eggs present opportunities to improve assumptions regarding association between egg output and number of worm pairs in an individual (Lu et al., 2010; Gower, Gehre, Marques, Lwambo, & Webster, 2017).

An additional complexity when considering mating probabilities in a hybrid system is that of mating preferences. Mating preference can be very challenging to determine even in experimental infection, and schistosome mating “choices” within hosts in hybrid zones are likely to be numerous and complex. Furthermore, it appears that schistosomes may not be monogamous as once thought, with evidence of competitive mating interactions between species, including the ability for males to take females away from existing pairings and increased polygamy under strong selective pressure (Webster, Gower, & Blair, 2004; Webster & Southgate, 2003; Webster, Southgate, & Tchuem Tchuente, 1999).

The challenge of incorporating mating preference into a worm burden framework has previously been considered by Morand and colleagues (Morand, Southgate, & Jourdane, 2002) and applied to another schistosome hybrid system, specifically the replacement of *S. intercalatum* (now *S. guineensis*) by *S. haematobium* and *S. intercalatum* with *S. haematobium* hybrids in Cameroon. This model assumed a mating preference for worms of the same species (i.e., *S. haematobium* will mate preferentially with *S. haematobium* and excess *S. haematobium* males will only mate with *S. intercalatum* if there are no available *S. haematobium* females).

Molecular techniques including multilocus approaches which utilize mitochondrial and nuclear markers (Huyse et al., 2009; Léger et al., 2016; Webster et al., 2013) can give indications as to whether hybrid progeny are the result of an early pairing or later backcrosses, as well the direction of the pairings. Such data could be used to inform and improve assumptions about mating preferences in a framework similar to that described by Morand and colleagues, for the more complex zoonotic hybrid system.

Whilst mate pairings are the primary parasite interaction of interest in the zoonotic hybrid schistosome system, other factors that may need to be incorporated into a model include the potential for some degree of cross‐immunity and/or within‐host competition for resources, although it is unknown whether or how such factors may play a role in a zoonotic hybrid schistosome system.

### 4.2 Multiple definitive hosts: hybrid schistosomes as part of a multihost system

Modelling multihost systems is recognized as posing many additional challenges over those of single‐host systems and necessitates some degree of disentangling if such models are to inform control measures (Webster, Borlase, & Rudge, 2017). Identifying the key hosts in the hybrid system is therefore one of the most important challenges as well as potentially one of the most informative aspects of a model of this system; if animal reservoirs were found to be key for maintaining transmission, then control strategies would need to target nonhuman animals to be effective.

One of the first considerations for modelling a multihost system is determining which host species to incorporate. In Figure 3, we have included the three livestock species known to be infected with *S. bovis,* although other potential zoonotic sources of infection may include wildlife species. Once the appropriate host species to be incorporated into the model have been identified, the next challenge is to characterize the multihost dynamics and identify the relative role of each host species in transmission. Conceptual frameworks have been proposed to identify which hosts are driving transmission within multihost systems (Fenton et al., 2015; Haydon, Cleaveland, Taylor, & Laurenson, 2002), classifying hosts as “maintenance” or “nonmaintenance” (depending on whether transmission can be maintained within that host species alone), and “essential” or “nonessential” (depending on whether transmission could persist without that host species).

Applying these concepts to the preliminary model framework in Figure 3, some assumptions regarding the role of certain hosts in transmission are already made based on current knowledge. For example, it is assumed here that livestock are not susceptible to and therefore are nonmaintenance hosts for, *S. haematobium* and its hybrids. Many questions regarding the role of different hosts in this system can however only be addressed through model parameterization and fitting to field data. Such questions include what is the relative contribution of each livestock species to *S. bovis* transmission? And, are humans maintenance hosts for the hybrid parasites after they have been generated from the initial cross‐species pairings, or is ongoing zoonotic spillover of *S. bovis* into humans required for the hybrids to persist? The latter would represent an interesting situation whereby livestock may be essential, but nonmaintenance hosts for the hybrid schistosomes.

In principle, parameterization and fitting of a multihost model to estimate *R*_0_ for each host species *i* (*R*_0*,i*_) and the system overall (*R*_0,tot_) can be used to assess the potential for transmission maintenance within each host species.

This has been illustrated in a model of the multihost *S. japonicum* system in China by Rudge et al. (2013). In that study, a prevalence framework was applied, and with available national surveillance data suggesting that dynamics had reached an approximately steady‐state equilibrium, *R*_0_ estimates could be derived from model fitting to cross‐sectional field data. However as noted previously, worm burden models are likely to be necessary for the zoonotic hybrid schistosome system, in order to incorporate the mating probabilities between different schistosome species. Here therefore, derivation of *R*_0_ values would rely on estimates of the number and distribution of worms in each host species in addition to prevalence estimates. Furthermore, the assumption of steady‐state dynamics would not necessarily be reasonable for all (or any) of the parasite species in the hybrid system. If, for example, the host switch which resulted in hybrids was relatively recent phenomena, it may be the system has not yet reached equilibrium, and in the example of Cameroon (Morand et al., 2002; Tchuem Tchuente et al., 1997), over time hybrids have come to replace their parent species. Longitudinal data on the prevalence and intensity of different schistosome species (and their hybrids) in the different host populations could be used to elucidate the stability of the system.

In contrast to the example of *S. japonicum* (which based on current evidence is typically modelled as a true generalist parasite with potential for bidirectional transmission between all available definitive host species), a model of a zoonotic hybrid schistosome system requires careful consideration regarding which interhost species transmission pathways are possible for each parasite type. Given the absence of evidence for *S. haematobium* or its hybrids successfully infecting the livestock population, there currently does not appear to be flow of transmission from humans to animals within the West African hybrid system. Therefore, in this case, the process potentially of most interest is the “spillover” of the animal schistosomes into the human population, represented by parameter *A*_*H*_ *,*_*Sb*_ in Figure 3. Estimating the frequency of such spillover events, the risk factors involved and the relative importance of the different potential animal sources (cattle, sheep, goats and possibly wildlife) could prove key to understanding the role of animals in the hybrid schistosome system.

Lloyd‐Smith et al. (2009) discussed the importance of spillover force of infection in emergence dynamics and proposed a general framework in which it can be defined as the product of the prevalence in reservoir, the reservoir–human contact rate and the probability of infection given contact. However, this framework cannot be easily applied to the hybrid schistosome system. Due to indirect transmission via snail intermediate hosts, transmission‐relevant contact rates between humans and reservoir sources of infection are difficult to measure. Additionally, when considering the probability of infection given contact, current evidence indicates that the probability infection of a human host with a livestock schistosome leads to a patent infection is dependent on that human host being currently infected with *S. haematobium* (excepting the occasional example of *S. bovis: S. curassoni* hybrids infecting humans (Léger et al., 2016)).

If the initial cross‐species pairings between animal and human schistosomes (*Sh:Sb* pairings in Figure 3) are therefore considered to represent the true spillover event of interest, the prevalence of human schistosomiasis (in addition to reservoir prevalence) and dispersion of worms in all populations would all need to be factored into estimating the probability of such events in this system. Here, then the capacity for molecular tools to identify F1 hybrid infections in humans could give scope to estimate the rate of spillover into the human population.

A further complicating factor in the hybrid system though is that progeny of cross‐species pairings which infect snails can subsequently re‐infect humans and then backcross with parent species. Whilst F1‐type hybrids can be identified with some confidence using currently available techniques, distinguishing “who mated with whom” in later‐generation hybrids becomes much less clear. More advanced molecular techniques such as whole‐genome sequencing may offer insights, but currently separating contribution of animal‐to‐human and human‐to‐human infections in maintaining transmission overall remains a considerable challenge.

Other model‐based approaches for differentiating zoonotic spillover from human–human transmission include those based on acute or outbreak infections for which timing of infections can be determined with some accuracy from case‐onset data (e.g., Kucharski et al., 2014). This is not currently possible for schistosomiasis, again highlighting the need for novel approaches such as model fitting to genotyped egg‐count data, in order estimate relative rates of zoonotic and human‐to‐human transmission.

Estimating the relative contribution of the different animal species to the force of infection in snail hosts (*B*_*C,Sb*,_ vs. *B*_*G,Sb*_ vs. *B*_*O,Sb*_ on the example shown in Figure 3), and their corresponding relative role in transmission to the human population, will require up‐to‐date estimates of the prevalence of schistosomiasis in each species, the size of each the animal population (*C*,* O* and *G* in Figure 3) in hybrid zones, and depending on the framework used, estimates of the worm burden and egg‐shedding rate in each animal population. Furthermore, given the highly focal nature of schistosome transmission, these parameters may vary significantly between areas. However, aside from the many complexities already discussed, basic data such as prevalence of schistosomiasis in animal populations in hybrid zones are currently lacking. Lack of surveillance data is a common problem when it comes to parameterizing any model including animal populations (Brooks‐Pollock, de Jong, Keeling, Klinkenberg, & Wood, 2015); although differing ethical constraints do mean that certain data and parameters are more easily obtainable from animals than from human populations. For example whilst it is unlikely that human autopsy work of Cheever et al., could be repeated now, in livestock species the number of worms per host and corresponding number of eggs shed could be estimated by sampling abattoir specimens (Cheever, 1968 & Cheever et al., 1977).

The need for new and improved diagnostics for detecting (and quantifying the intensity of) helminth infections was identified as a research priority by the Disease Reference Group on Helminth Infections (DRG4) (Boatin et al., 2012; Utzinger, 2012), for purposes of monitoring and evaluation of control programmes, assessment of drug efficacy and mathematical modelling (McCarthy et al., 2012). Lack of sensitivity amongst human schistosomiasis diagnostic tests that rely on egg detection is well documented (Kongs, Marks, Verlé, & Van Der Stuyft, 2001; Savioli, Hatz, Dixon, Kisumku, & Mott, 1990; Warren, Siongok, Houser, Ouma, & Peters, 1978; Yu, de Vlas, Jiang, & Gryseels, 2007), and in West Africa, sensitivities of such tests in animals are unquantified but potentially even lower (Olaechea, Christensen, & Henriksen, 1990). In recent years, use of the point‐of‐care circulating cathodic antigen (CCA) urine test in humans has become widespread and is considered to represent higher test sensitivity compared to other field tests (Kittur, Castleman, Campbell, King, & Colley, 2016; Mwinzi et al., 2015; Ochodo et al., 2015). However, data on how well this test performs in animals, and indeed for hybrid schistosome species, are currently lacking. Such a lack of field diagnostic tests for animal schistosomiasis, validated in the West Africa setting, represents a considerable challenge for parameterizing the animal components of a hybrid system model. A further consideration for the hybrid system is the need to differentiate between different schistosome species (and hybrids thereof) being shed by individuals of each definitive host species. This currently means applying diagnostic tests that enable the collection of miracidia or eggs for molecular analysis. Such tests have been described and successfully used for both human and animal schistosomiasis (Gower et al., 2007; Rudge et al., 2009), and should be incorporated when planning data collection for the purposes of parameterizing a model of the hybrid system.

### 4.3 An evolving pathogen: hybrid schistosomes as a novel and evolving parasite

Pathogen evolution presents many challenges for infectious disease modelling, with Metcalf and colleagues (Metcalf et al., 2015) identifying several key challenges, all of which are highly pertinent to the zoonotic hybrid schistosome system. These include defining and measuring parasite fitness (with *R*_0_ often used as an indicator of this), capturing the impact of co‐infection and host–parasite interactions, understanding maintenance of genetic diversity, and modelling the impact of genetic inheritance and exchange.

Many epidemiological models have relied on the a priori assumption that a pathogen population is homogenous and unchanging, but there is growing recognition of the limitations of such assumptions in systems that are subject to strong evolutionary pressures (Gandon et al., 2016). For example, most schistosomiasis models incorporate control measures such as chemotherapy by assuming a single, fixed reduction in worm population (impacting overall worm loss rate, *μ*_*H,Sh*_ in the schematic in Figure 2) (Barbour, 1996; Gurarie & King, 2014; Woolhouse, 1991), but in the hybrid system, the possibility of differential drug efficacy in hybrids must be considered. Similarly, laboratory studies have indicated the potential for schistosome hybrids to exhibit traits such as increased worm pair fecundity and increased cercarial shedding (Taylor, 1970; Webster & Southgate, 2003). This highlights the need for further empirical studies, for example comparing praziquantel responses and fecundity of the different schistosome genotypes in hybrid zones. However, the relationship between such observable traits and reproductive success is not necessarily straightforward, with number of new hosts infected often also depending on frequency of other genotypes both in the individual host and in the system as a whole (Metcalf et al., 2015).

In addition to the presence of hybrids potentially impacting on effectiveness of control measures, the evolutionary pressure of interventions may likewise influence the hybrid system. Conceivable impacts include the possibility of reduced praziquantel efficacy (as has been observed in Uganda, (Crellen et al., 2016)), and the potential for an increasing role of animals in maintaining transmission as infection levels in the human population decrease (as may have been the case in the example of Guinea Worm, (Callaway, 2016; Eberhard et al., 2014)).

Developing a model which adequately describes the underlying mechanisms of genetic inheritance, and exchange is also a major challenge in the zoonotic hybrid system. Due to introgression, sequential generations originating from an *Sh:Sb* hybrid worm, for example, may become increasingly similar (both genetically and phenotypically) to a “pure” *S. haematobium* worm. This would be difficult to incorporate in a framework modelling discrete parasite types (such as that in Figure 3) without some way of tracking parasite lineages across multiple generations. An alternative, but even more complex, approach might involve modelling a manageable number of hypothetical parasite genes (e.g., each with *Sh* and *Sb* alleles) to capture mechanisms of genetic recombination and inheritance, thus allowing some “fluidity” of worms between the parasite types in the system.

## 5 MODELLING OPPORTUNITIES: CONTROL OF SCHISTOSOMIASIS IN HYBRID ZONES

Having described the many diverse and complex challenges of developing and parameterizing a model of the hybrid schistosome system, it must be remembered that the role of a mathematical model is not to faithfully reproduce every single aspect of a disease system, and indeed attempting to do so is unlikely to be informative. Modelling is a highly iterative process, and even the process of conceptualizing a model of a complex system such as the hybrid system can force us to focus research questions and refine hypotheses, as well as help to clarify assumptions and identify parameters which are critical to the dynamical behaviour of the system, even when the numerical range of parameters is unknown (Heesterbeek & Roberts, 1995). Table 2 summarizes the key challenges for modelling the zoonotic hybrid schistosome system identified in this paper and possible approaches to address these. The wide range of empirical data needs identified here illustrates the importance of modellers being fully integrated into interdisciplinary teams tackling helminthic diseases and involved with the planning of data collection from the outset (Basáñez et al., 2012). Further insights into where additional measurements or improved confidence intervals are needed could be gained through identifiability analysis (Raue et al., 2009). Even without complete data for all the potential nonhuman hosts that may enable full parameterization of a multihost model and thus, for example, estimation of each hosts’ contribution to *R*_0_, analytical and numerical solutions of models could still give insights into the importance of the animal reservoir, as well as the relative importance of other parameters such as mating probability and egg‐shedding rates. Model simulations could also be used for various scenario analyses to gain at least qualitative or semi‐quantitative insights into how perturbations to the system, such as increasing livestock densities or chemotherapeutic interventions targeting livestock populations, may affect the hybrid dynamics. Given the complexity of the system, the impact of such selective pressures on model outputs may not always be intuitive.

**Table 2:** Key challenges for mathematical modelling of zoonotic hybrid schistosome systems in sub‐Saharan Africa, and potential solutions and research priorities to address these

Challenge	Potential solutions and research priorities
Incorporating multiple schistosome species and their hybrids	Extension of existing (e.g., worm burden) model frameworks to incorporate multiple schistosome genotypes and hybridization mechanisms Use of diagnostic tests that enable collection of molecular material which permit subsequent laboratory analysis to distinguish schistosome genotypes Use of multilocus techniques that enable identification of sexual direction of hybridization
Incorporating multiple definitive host species	Epidemiological studies in livestock and wildlife populations Sampling to include both faeces and urine specimens from livestock species Adaptation and validation of existing diagnostic tests for animal schistosomes in Africa
Necessity for model to incorporate heterogeneities and mating interactions between schistosome species and their hybrids	Model structure to differentiate between schistosome genotypes and incorporate mating probabilities, for example worm burden framework
Parameterizing multihost transmission dynamics and estimating zoonotic spillover	Collection of field data on livestock population densities and egg‐shedding rates Use of currently available laboratory techniques to identify F1‐type hybrids, for example multilocus techniques Further development of laboratory techniques such as whole‐genome sequencing, to better track “who acquires infection from whom”
Characterizing heterogeneities in “fitness” traits that may influence transmission rates of hybrids vs “pure” schistosome species and how these traits may be changing in response to evolutionary pressure	Empirical studies to compare biological traits (e.g., cercariae‐shedding rates by snails and egg reduction rate postpraziquantel treatment in definitive hosts) between schistosome genotypes
Quantification of spillover contribution of different livestock species	Field data to include egg‐shedding rate per livestock host, livestock population estimates

Modelling studies have previously been used to explore the factors influencing spread of anthelmintic resistance genes in parasite populations, including schistosomes, in response to chemotherapeutic pressure (Castillo‐Chavez, Feng, & Xu, 2008; Churcher & Basáñez, 2008; Feng, Curtis, & Minchella, 2001). Resistance to anthelmintics used in livestock has been reported for many years (Geurden et al., 2015; Kaplan, 2004; Waghorn, Miller, & Leathwick, 2016; Wolstenholme, Fairweather, Prichard, Von Samson‐Himmelstjerna, & Sangster, 2004), and a major concern regarding any chemotherapeutic intervention targeting the animal population is that this may risk praziquantel resistance developing and spreading to the human schistosome population. Here, again modelling and understanding the factors influencing the probability of spillover, in particular spillover with ongoing transmission, could play an important role. Although currently no genes specifically conferring resistance to praziquantel have yet been identified (Wang, Wang, & Liang, 2012), a multihost framework incorporating spillover could be used to explore the probability of a theoretical gene that is widespread in the animal schistosome population becoming widespread in the human population.

## 6 CONCLUSIONS

The complexity of multihost multiparasite systems challenges our ability to understand their ecological and evolutionary dynamics and design appropriate strategies for their control. Evidence for hybridizations and introgressions between co‐infecting parasite species is gathering, in line with improved molecular diagnostics and genome sequencing techniques capable of detecting such phenomena. Global changes including human and animal migration, combined with factors such climate change and habitat modifications, will provide increasing opportunities for such hybridization and introgressions, with potential implications for the further emergence, spread and evolution of novel and virulent strains.

Mathematical modelling provides a crucial tool for improved understanding of complex systems such as that of zoonotic hybridized parasites, including informing priorities for data collection, diagnostics and laboratory studies, generating and testing hypotheses, and exploring the impact that hybridizations may have on control measures, as well the impact that evolutionary pressures including control measures may have on driving the emergence and spread of parasite hybrids. The case example of zoonotic hybrid schistosomes of the haematobium group in West Africa represents a valuable and unique opportunity for empirical investigation into the potential impact of hybridization and introgressions in a multihost system of significance for public and veterinary health. Through examination of existing schistosomiasis modelling frameworks and considering how such frameworks could be extended for the multihost hybrid schistosome system in West Africa, key questions and knowledge gaps have been identified, including, for example, how field data and currently available molecular techniques can be best combined to infer the risk of spillover from the animal to the human population.

Addressing these challenges presents many conceptual and practical obstacles, but developing a mathematical model of the zoonotic hybrid schistosome system would represent an important and novel contribution, not only to inform the design of control and elimination strategies in this system, but also for advancing and exploring the theory of epidemiological–evolutionary dynamic interactions in multihost, multipathogen systems more generally.

## ETHICS

This work involved no human subjects.

## AUTHORS’ CONTRIBUTIONS

All authors contributed substantively to the manuscript.

## COMPETING INTERESTS

The authors have no competing interests.

## DATA ARCHIVING

This manuscript presents no novel data.
